# Mental health clinician attitudes to the provision of preventive care for chronic disease risk behaviours and association with care provision

**DOI:** 10.1186/s12888-016-0763-3

**Published:** 2016-03-02

**Authors:** Kate Bartlem, Jenny Bowman, Kate Ross, Megan Freund, Paula Wye, Kathleen McElwaine, Karen Gillham, Emma Doherty, Luke Wolfenden, John Wiggers

**Affiliations:** Population Health, Hunter New England Local Health District, Booth Building, Wallsend Health Services, Longworth Avenue, Wallsend, NSW 2287 Australia; School of Psychology, Faculty of Science and Information Technology, University of Newcastle, University Drive, Callaghan, NSW 2308 Australia; Hunter Medical Research Institute, Clinical Research Centre, Lot 1 Kookaburra Circuit, New Lambton Heights, NSW 2305 Australia; School of Medicine and Public Health, Faculty of Health and Medicine, University of Newcastle, University Drive, Callaghan, NSW 2308 Australia

**Keywords:** Mental illness, Preventive health care, Attitudes, Health behaviour, Psychiatric

## Abstract

**Background:**

Preventive care for chronic disease risk behaviours by mental health clinicians is sub-optimal. Little research has examined the association between clinician attitudes and such care delivery. This study aimed to explore: i) the attitudes of a multi-disciplinary group of community mental health clinicians regarding their perceived role, perception of client interest, and perceived self-efficacy in the provision of preventive care, ii) whether such attitudes differ by professional discipline, and iii) the association between these attitudes and clinician provision of such care.

**Method:**

A telephone survey was conducted with 151 Australian community mental health clinicians regarding their attitudes towards provision of assessment, advice and referral addressing smoking, nutrition, alcohol, and physical activity, and their reported provision of such care. Logistic regression was used to examine the association between attitudes and care delivery, and attitudinal differences by professional discipline.

**Results:**

Most clinicians reported that: their manager supported provision of preventive care; such care was part of their role; it would not jeopardise their practitioner-client relationships, clients found preventive care acceptable, and that they had the confidence, knowledge and skills to modify client health behaviours. Half reported that clients were not interested in changing their health behaviours, and one third indicated that the provision of preventive care negatively impacted on time available for delivery of acute care. The following attitudes were positively associated with the provision of preventive care: role congruence, client interest in change, and addressing health risk behaviours will not jeopardise the client-clinician relationship.

**Conclusions:**

Strategies are required to translate positive attitudes to improved client care and address attitudes which may hinder the provision of preventive care in community mental health.

## Background

People with a mental illness experience poorer physical health than the general population and markedly lower life expectancy as a consequence [1, 2]. A greater prevalence of chronic disease risk behaviours, including tobacco smoking, inadequate nutrition, harmful alcohol consumption, and physical inactivity contribute substantially to this health inequity [1, 2]. The prevalence of such behaviours among people with a mental illness varies substantially by diagnosis and setting. Recent Australian data identified that among community mental health clients, 96 % were at risk for at least one of these four behaviours, with risk highest for inadequate nutrition (87 %), followed by tobacco smoking (51 %), inadequate physical activity (47 %) and harmful alcohol consumption (43 %) [3].

In addition to the impact of such behaviours on the physical health of people with a mental illness, a growing body of research demonstrates that reducing chronic disease health risk behaviours for people with a mental illness can positively impact on their mental health outcomes [4–7]. Lifestyle or behaviour change interventions have been demonstrated to be effective in assisting people with a mental illness to improve their health risks behaviours and physical health more broadly [8–11]. Such lifestyle interventions have further been shown to positively impact mental health outcomes, including reducing psychiatric symptoms [4, 12, 13]. Mental health services are recommended to provide care that seeks to modify such health risk behaviours [14–18], and may provide a particularly opportune setting for addressing these risks due to the availability of multidisciplinary teams with a wide range of relevant skills and expertise, and the often frequent and ongoing nature of care provided [19]. Despite these recommendations and the benefits of such care, preventive care is not routinely provided [20–23]. For example, in a study of 1,610 psychiatrists in the USA, 6 % of clients were reported to be provided diet counselling, 4 % exercise counselling, and 12.4 % smoking-cessation counselling [22]. Given the suboptimal provision of such care, analysis of the determinants of such care practices is required.

In general health services, factors suggested to impede the provision of preventive care have included attitudes that provision of such care is not an appropriate role of clinicians, perceptions that clients are not interested in changing their health risk behaviours, and a lack of clinician self-efficacy in providing preventive care (skills, knowledge, confidence and perceived effectiveness) [24, 25]*.* Few studies have examined the impact of mental health clinician attitudes on the provision of care addressing client physical health risk behaviours [26, 27]. In one such study of the attitudes and practices of Canadian community mental health care workers towards smoking cessation care, a belief that there was sufficient time in a consultation to address tobacco use, that tobacco cessation care was a part of their role, greater confidence in providing smoking cessation care, and a perception of clients being interested in stopping smoking were positively associated with the self-reported provision of smoking cessation care [26]. Similarly, a survey of UK psychiatric inpatient and community mental health nurses found that positive attitudes towards the role of nurses in providing physical health care (including addressing health risk behaviours), and greater confidence in delivering such health care were positively associated with self-reported delivery of such care [27].

The prevalence of such attitudes have been reported to vary among mental health clinicians [28–32], with for example, support for the provision of smoking cessation care reported to vary between 43 % and 87 % across studies [28–30, 32]. Similarly variable findings (23 % and 77 %) have been reported regarding mental health clinician perception of client interest in receiving smoking cessation care [31, 32]. In the USA, 90 % of psychiatrists expressed confidence in their ability to advise clients of the risks of smoking, but only 34 % in referring clients to ongoing cessation care [31].

The attitudes of mental health clinicians to the provision of preventive care for behavioural risks other than smoking have been addressed in only a limited number of studies [27, 33, 34]. Two studies in the United Kingdom have reported high levels of clinician support for providing such care regarding nutrition (78 %-92 %), physical activity (76 %-95 %), and alcohol consumption (83 %-92 %) [27, 33]. With regard to clinician reported self-efficacy, approximately one quarter of inpatient nurses (23 %-38 %) reported a lack confidence regarding the provision of preventive care for nutrition, physical activity, and smoking [33]. In a third qualitative study, Australian community mental health managers reported that their ‘core business’ was to assess and treat mental illness, with physical health related issues seen to be of ‘secondary importance’ [34].

Given the limited scope and variable findings of studies regarding mental health clinician attitudes to the provision of care addressing the prevention of chronic disease risk behaviours, a study was undertaken to investigate: i) the attitudes of a multi-disciplinary group of community mental health clinicians regarding their perceived role, perception of client interest, and perceived self-efficacy in the provision of such care, ii) whether such attitudes differ by professional discipline, and iii) the association between these attitudes and clinician provision of such care.

## Methods

### Design and setting

A cross-sectional survey of clinicians working within community mental health services was undertaken across one local health district in New South Wales, Australia (May to August, 2010). The district has a population of approximately 850,000 residents across both urban and rural communities. Five months prior to the survey, the district introduced a policy requiring the provision of preventive care addressing chronic disease risk behaviours to all community mental health service clients [35]. Ethics approval was obtained from the Hunter New England (approval No. 09/06/17/4.03) and University of Newcastle (approval No. H-2010-1116) Human Research Ethics Committees. The Hunter New England Human Research Ethics Committee approval included permission to access the electronic administrative record system.

### Sample

Nineteen community mental health services in the district provided the following forms of care to adult clients: older person’s care, psychiatric rehabilitation, early diagnosis, neuropsychiatry, comorbid mental health and substance use, eating disorders and borderline personality disorder services. The services were staffed by multi-disciplinary teams including nurses, psychologists, psychiatrists, social workers, and occupational therapists. Care was provided to clients following either a psychiatric facility inpatient stay, a referral from a GP or other provider, or self-referral.

All clinicians in the 19 services were eligible to participate in the study if they had been employed by the service for at least three months and provided care to a minimum of 10 clients in the prior two months. Eligible clinicians were identified from an electronic administrative record system.

### Recruitment and data collection

Eligible clinicians were mailed an information letter and subsequently telephoned during work hours to participate in a 20 minute computer assisted telephone interview. The survey was developed for the purpose of this study, as the authors were not aware of any validated measures for assessing the provision of preventive care, or attitudes towards doing so, among mental health clinicians. Interview items were based on the findings of previous studies of clinician reported barriers to the provision of preventive care in both general and mental health services [24, 25, 27–33], and reported preventive care provision [36]. The survey was pilot tested with community health clinicians and administered by trained interviewers.

### Measures

#### Clinician characteristics

Clinicians were asked their: age (years), employment (full time, part time, casual, other), and length of professional employment (years). Additional information collected from the electronic administrative record system for consenters and non-consenters included: gender, Aboriginal and/or Torres Strait Islander origin, and professional discipline (nurse, psychiatrist/other medical, psychologist, occupational therapist, social worker, other).

#### Provision of preventive care

Preventive care was assessed with respect to clinician provision of three elements of care: assessment, brief advice, and referral/arranging ongoing support [37–39] regarding four health risk behaviours: smoking, inadequate nutrition, harmful alcohol consumption, and physical inactivity. Fruit and vegetable under consumption were selected as the indicator for inadequate nutrition due to their contribution to the chronic disease burden [40], the resulting emphasis on such consumption in the national guidelines, and evidence of a protective effect against cardiovascular diseases, diabetes, and some cancers [41–43].

Participants were asked to estimate the proportion (0-100 %, don’t know) of all new adult clients for whom they assessed smoking status; fruit and vegetable consumption; alcohol consumption; and physical activity levels in the past two months. For those patients assessed as being at-risk for each behaviour, participants were asked to estimate the proportion that they had advised to modify that risk behaviour, and the proportion to whom they provided referral/follow-up. Full methods and results regarding the delivery of preventive care have been published previously [23]. The current study presents a summarised version of this data only as the basis for examining the association between clinician attitudes and the delivery of such preventive care.

#### Clinician attitudes regarding delivery of preventive care

Five-point Likert scale items (strongly agree to strongly disagree) were used to assess clinician attitudes regarding:

*Perceived role in provision of preventive care:* level of agreement with five statements regarding mental health clinicians’ role in providing preventive care (Table 1).

**Table 1:** Clinician reported role congruence and client interest in preventive care for all four behavioural health risks combined: % (n) agree/strongly agree

Attitudinal Item	% (n)
Role Congruence	
1. My manager believes the provision of preventive care is important.	87.4 % (132)
2. It is part of my role to provide preventive care to clients.	90.7 % (137)
3. Addressing health risk behaviours won’t jeopardise my relationship with the client.	86.1 % (130)
4. Providing preventive care for health risk behaviours leaves me time to provide acute care.	66.2 % (100)
5. Clients find it acceptable for me to talk with them about their health risk behaviours.	92.7 % (140)
Client Interest	
6. Clients I see are interested in changing their health risk behaviours	47.7 % (72)

*Perception of client interest in modifying health risk behaviours:* level of agreement with a statement addressing perceived client interest in improving their health risk behaviours (Table 1).

*Self-efficacy in providing preventive care:* level of agreement with four statements addressing perceived ability to provide preventive care for each of the four health risk behaviours (Table 2).

**Table 2:** Clinician reported self-efficacy regarding the provision of preventive care for four health behaviour risks: % (n) agree/strongly agree

Attitudinal Item	% (n)
1. I feel confident to talk with clients about their health risk behaviours.	
Smoking	96.7 % (146)
Inadequate nutrition	96.0 % (145)
Alcohol	97.4 % (147)
Physical inactivity	98.0 % (148)
All behaviours^a^	92.7 % (140)
2. I have the knowledge and skills to provide preventive care to clients regarding health risk behaviours.	
Smoking	95.4 % (144)
Inadequate nutrition	90.1 % (136)
Alcohol	93.4 % (141)
Physical inactivity	95.4 % (144)
All behaviours^a^	88.1 % (133)
3. There are services to which I can refer clients to change their health risk behaviours	
Smoking	91.4 % (138)
Inadequate nutrition	82.1 % (124)
Alcohol	92.7 % (140)
Physical inactivity	80.1 % (121)
All behaviours^a^	72.2 % (109)
4. Clients will change their health risk behaviours because of the care I provide	
Smoking	86.1 % (130)
Inadequate nutrition	86.8 % (131)
Alcohol	88.1 % (133)
Physical inactivity	90.7 % (137)
All behaviours^a^	76.2 % (115)

^a^All behaviours variable reflects clinicians who responded ‘agree’ or ‘strongly agree’ to the item for all four health risk behaviours

### Statistical analyses

Analyses were conducted with SPSS V19 and SAS analysis package (SAS, V9.3). Chi squared analyses were used to compare consenters and non-consenters regarding gender, Aboriginality and professional discipline. Responses to all attitudinal items were collapsed into two categories: either strongly agree/agree or unsure/disagree/strongly disagree. For each self-efficacy item, an ‘all behaviours’ variable was calculated to reflect responses regarding perceived ability to provide care for all four of the health risk behaviours. Responses to each preventive care provision item were condensed to reflect the proportions of clinicians who provided care to 0-79 % of clients (including responses of ‘don’t know’), and clinicians who provided care to 80-100 % of clients [36, 44]. For each element of care, variables were calculated to examine care provided to 80 % or more of clients for all behaviours combined (‘all behaviours’). Care outcomes were dichotomised in order to examine attitudinal associations with care being provided at an ‘optimal’ level. Optimal care was defined as care provided to 80 % or more of clients, based on previous research [36, 44] Descriptive statistics were used to examine clinician characteristics, attitudes, and the provision of preventive care.

#### Attitudinal differences by professional discipline

To examine whether reported attitudes differed by professional discipline (nurse [reference group]; allied health: psychologist, social work, occupational therapy, other; psychiatrist/other medical), separate binomial regression analyses were undertaken for each attitudinal item (10 models). For items regarding self-efficacy, regression analyses were conducted for both the ‘all behaviours’ variable, and the self-efficacy items for each behaviour separately.

#### Association between clinician attitudes and provision of preventive care

Chi-squared analyses were initially undertaken to examine the association between each clinician attitude (agree/strongly agree versus unsure/disagree/strongly disagree) and the provision of each form of care (0-79 % versus 80-100 %). Attitudinal items found to be associated with each form of care at *p* < .25 [45] were entered into separate logistic regression models for each care outcome, using a backwards stepwise process until all variables in the model remained significant (15 models) (*p* < .05). Self-efficacy items directly related to the specific outcome being examined were entered for each model. For instance, self-efficacy items related to smoking were entered for the smoking care model, while the self-efficacy for all behaviours combined items were entered into the care for ‘all behaviours’ models. The logistic regression models controlled for the effects of clinician age, gender, length of professional employment, remoteness of service, and professional discipline, as previous research has demonstrated an association between these variables and the provision of preventive care [36, 46]. Collinearity diagnostics were used to examine the presence of collinearity between the attitudinal variables in the final models.

## Results

### Clinician characteristics

Of 195 identified clinicians, 170 (87.2 %) were eligible to participate. Of these, 151 (88.9 %) completed the survey. No significant differences were identified between consenters and non-consenters. The majority of participants were female (58.3 %), aged between 20–49 years (56.7 %) and not of Aboriginal or Torres Strait Islander origin (96.7 %). Most participants were registered nurses (42.4 %; allied health 35.7 % [comprised of psychologists 13.2 %, occupational therapists 7.3 %, social workers 8.6 %, and other allied health 6.6 %); psychiatrist/other medical 21.9 %), employed in full time work (71.5 %) and reported working in their profession for over five years (87.5 %).

### Provision of preventive care

A previously published paper has presented this data regarding the provision of preventive care [23]. In summary, the paper reported that the proportion of clinicians who reported providing preventive care to 80-100 % of their clients ranged from 13.2 % (fruit and vegetable consumption) to 89.4 % (alcohol consumption) for assessment; 46.3 % (fruit and vegetable consumption) to 80.1 % (alcohol) for advice, and 22.5 % (fruit and vegetable consumption) to 60.9 % (alcohol) for any type of referral. The following proportions of clinicians reported providing preventive care to 80-100 % of clients for all four behaviours: 8.6 % (assessment), 25.2 % (advice), 9.9 % (referral). For full findings refer to the original Elsevier published data [23].

### Attitudes toward provision of preventive care

#### Perceived role in providing preventive care

Over 86 % of participants reported that: care was part of their role, and that providing such care would not jeopardise the client-clinician relationship. Almost all (93 %) reported that clients found it acceptable to discuss their health risk behaviours. One third of participants indicated that providing such care may detract from time available for delivery of acute care (Table 1). Compared to nurses, psychiatrists and other medical practitioners were less likely to report that the provision of preventive care for all risk behaviours was part of their role (OR 0.221, *p* = .043, 95 % CI 0.051 - 0.951) and that providing preventive care for all behaviours left sufficient time to provide acute care (OR 0.368, *p* = .025, 95 % CI 0.154 - 0.882).

#### Perception of client interest in changing health risk behaviours

Less than half (47.7 %) of all participants agreed or strongly agreed that clients were interested in improving their health risk behaviours (Table 1). No differences in responses were identified between professional groups in terms of perceived client interest in modifying their health risk behaviours.

#### Self-efficacy

Over 88 % of all participants agreed or strongly agreed that they had the confidence, knowledge and skills to provide preventive care for all four health risk behaviours, and 76 % agreed or strongly agreed that clients would change all of their behaviours in response to such care (Table 2). Over 72 % of participants agreed or strongly agreed that referral services were available to which they could refer clients for all behaviours, with referral services for nutrition and physical inactivity seen to be the least available. No differences were identified between professions regarding reported self-efficacy in providing preventive care for all four risk behaviours combined, or for any of the four behaviours separately.

#### Association between clinician attitudes and provision of preventive care

No attitudinal items were significantly associated with the provision of assessment for any of the four behaviours. Clinicians with a positive attitude towards their role in providing preventive care were more likely to provide advice regarding smoking (OR 6.1), fruit and/or vegetable consumption (OR 5.5), and physical inactivity (OR 3.6). Those who thought clients were interested in changing their health risk behaviours were more likely to provide advice for fruit and/or vegetable consumption (OR 2.2). Clinicians who reported that clients find it acceptable to talk with them about their health risk behaviours were less likely to provide advice for smoking (OR 0.2) and all four behaviours (OR 0.2) (Table 3).

**Table 3:** Association between clinician attitudes and the provision of preventive care to 80-100 % of clients^a,b^

Predictor^c^	B	SE	OR	95 % CI		*p*
**Advice to 80** %-**100** % **of at**-**risk clients**						
**Smoking** ^**d**^						
It is part of my role to provide preventive care to clients	1.8	0.7	6.1	1.5	24.8	*.* ***01***
Clients find it acceptable for me to talk with them about their health risk behaviours	−1.8	0.9	0.2	0.03	0.9	*.* ***03***
**Fruit and**/**or vegetable** ^**d**^						
It is part of my role to provide preventive care to clients	1.7	0.8	5.5	1.1	26.8	*.* ***04***
Clients I see are interested in changing their health risk behaviours	0.8	0.4	2.2	1.1	4.5	*.* ***03***
**Physical Activity**						
It is part of my role to provide preventive care to clients	1.3	0.6	3.6	1.1	12.4	*.* ***04***
**All Behaviours**						
Clients find it acceptable for me to talk with them about their health risk behaviours	−1.7	0.7	0.2	0.04	0.7	*.* ***01***
**Referral to 80** %-**100** % **of at**-**risk clients**						
**Alcohol**						
Addressing health behaviours won’t jeopardise my relationship with the client	1.2	0.5	3.2	1.2	9.0	*.* ***03***

^a^Logistic regression models adjust for clinician age, gender, length of professional employment, remoteness of service, and professional discipline

^b^Final logistic regression models unable to be calculated for fruit and/or vegetable assessment and all behaviours assessment as there were zero observations which provided care to 80-100 % of clients and who responded ‘unsure/disagree/strongly disagree’ to the attitudinal items entered

^c^The following outcomes had no significant associations with attitudinal variables hence are not presented in the table: assessment: smoking, fruit and/or vegetable, alcohol, physical activity, all behaviours; advice: alcohol; referral: smoking, fruit and/or vegetable, physical activity, all behaviours

^d^Collinearity diagnostics for smoking advice model and fruit and/or vegetable advice model: Variance of inflation = 1.0 and 1.01 respectively, indicating that collinearity was not present

The only referral outcome associated with an attitudinal item was alcohol. Clinicians who thought that addressing health behaviours wouldn’t jeopardise their relationship with their clients were three times more likely to provide a referral/follow-up for alcohol consumption (OR 3.2) (Table 3).

## Discussion

This study found a substantive majority of community mental health clinicians considered that the provision of care to prevent four chronic disease health risk behaviours was congruent with their role, and that they had sufficient knowledge, skills and resources to provide such care. Notwithstanding these positive findings, up to a third of clinicians considered that the provision of such care might negatively impact on delivery of acute care, one fifth were not aware of referral services for inadequate nutrition and physical inactivity, and more than half did not believe their clients were interested in changing their health risk behaviours. For the majority of attitudes, no differences were evident between professional disciplines. Positive associations with some forms of preventive care provision were identified. Strategies that strengthen these perceptions are required if the benefits of preventive care are to be maximised for all clients.

The finding that approximately half of participants reported that clients were not interested in changing their health risk behaviours is consistent with the findings of previous research [29, 31, 47]. For example, Australian psychiatric inpatient nurses have reported that their decision to provide smoking cessation care is primarily influenced by perceived patient receptivity [29]. Such selectivity in care provision contradicts care guidelines regarding provision of preventive care on a universal basis, and suggests additional strategies such as prompts and reminders may be required to facilitate clinician provision of preventive care to all clients [48]. Other studies have indicated such views of clinicians may be unfounded, with people with a mental illness being shown to be receptive to receiving preventive care and interested in improving their health risk behaviours [49–53]. Training and the dissemination of education resources has been found to positively impact primary care nurses’ misconceptions regarding physical health care for clients with a mental illness [54], and the current clinician misperceptions suggest a need for additional strategies to address possible deficits in clinician understanding of client needs in this regard.

Nearly one quarter of clinicians surveyed reported that the provision of preventive care impacted on the time available for the delivery of acute care, a perception commonly reported in studies across health services generally [24, 25] and mental health services specifically [27, 30, 31]. To address such concerns, models of preventive care provision have been developed to limit the amount of clinical consultation time required for its delivery. For example, the recommended 5A’s behavioural counselling framework [55] has been reduced to include only three elements of care: ‘assess, advise, and refer’ [37–39] thereby reducing time demands on the clinician during the consultation [38, 56]. Similarly, practice aids such as prompts, decision-aids, recording and automated referral protocols have been demonstrated to be both effective in enhancing the provision of preventive care and in reducing the time required of clinicians [48, 57, 58].

Recommended models of preventive care provision emphasise the importance of referral and/or follow up care [37–39]. In the current study, approximately one quarter of clinicians reported a lack of services to refer their clients to for behaviour change support; a finding that is reflective of previous research with psychiatrists [31]. Such a finding contrasts with the ready availability of free evidence-based health risk behaviour telephone services in the study area: Quitline for smoking cessation (www.icanquit.com.au/further-resources/quitline) and a telephone coaching service for addressing inadequate nutrition and physical inactivity (www.gethealthynsw.com.au). Despite the availability of such services and mechanisms to enable clinician referral of clients, research indicates under-referral to such services by clinicians [21, 59]*.* Clinician training may serve to increase awareness and utilisation of specialist prevention referral options [60]*.*

Few differences in attitudes regarding the provision of preventive care were identified between professional disciplines. However, psychiatrists were least likely to hold positive attitudes towards such care provision. Differences between study settings may account for the contrast between these findings and those from a UK inpatient setting [30] where no differences between medical practitioners and non-medical clinicians were identified. The current findings suggest that psychiatrists working in community mental health may benefit from training and additional evidence-based tools to support the development of more positive attitudes, given the importance of their leadership role in mental health services.

A number of attitudes were positively associated with some forms of preventive care, including the belief that providing preventive care was congruent with their role, that clients were interested in changing their health behaviours, and that addressing health risk behaviours would not jeopardise the client-clinician relationship. The finding of a negative association between clinicians reporting that clients find it acceptable to talk to them about their health risk behaviours, and the provision of some forms of preventive care is difficult to interpret. Although only speculative, it is possible that clinicians who perceive their clients to find such discussions acceptable are less likely to proactively engage with clients as they expect the client to initiate such discussions. The positive association results are consistent with previous research undertaken within Canadian community mental health care workers [26] and UK psychiatric inpatient nurses [27], whereby attitudes regarding role congruence, confidence in care provision, and client interest were associated with the self-reported provision of preventive care. Training and educational resources have been found to improve clinician attitudes and confidence towards providing physical health care to people with a mental illness [54, 61], and the current results suggest that such strategies addressing negative attitudes may be required to increase preventive care provision.

Despite the study findings suggesting that the large majority of community mental health clinicians are positively predisposed to providing preventive care, the prevalence of such care provision has been reported to be sub-optimal [20, 21, 23, 59]. Such a contradiction suggests that a gap exists between clinician attitudes and their professional practice; a gap that requires the implementation of additional practice change strategies if the intended benefits of international, national, and health service level preventive care guidelines [35, 62, 63] are to be realised.

Research evidence supports the use of a variety of strategies in facilitating clinical practice change, including clinical leadership and consensus, enabling systems and procedures, training and support, and monitoring and feedback [64–67]. It remains to be tested whether such organisational factors can increase the provision of preventive care within the community mental health setting. The study was undertaken within one local health district within one state in Australia, with a mandatory policy regarding the provision of preventive care to community mental health clients. It is unknown to what extent this policy may have impacted on clinician’s attitudes towards the provision of preventive care, hence, the generalizability of findings to other regions, jurisdictions or nations is unknown. The prevalence of preventive care delivery was self-reported by clinicians, and as such may have been influenced by demand characteristics and may not reflect actual care provided. Further, due to the relatively small number of psychologists, occupational therapists and social workers, the study examined the attitudes and practices of these allied health clinicians as a group. Future research should consider examining whether the preventive care attitudes and practices differ between different allied health disciplines.

## Conclusions

This study is among the first to examine the attitudes of multidisciplinary mental health clinicians regarding the delivery of preventive care with a focus on multiple health risk behaviours. Community mental health clinicians were generally positive towards providing preventive care, and a number of clinician attitudes were associated with an increased likelihood of preventive care provision. A number of possible barriers to the provision of preventive care were identified, including the belief that its provision might impact negatively on the delivery of acute care, clinicians not being aware of services to refer their clients to, and a perception that clients are not interested in changing their health risk behaviours. These findings provide a basis for future research regarding strategies to improve negative attitudes and translate the positive attitudes to improved client care.

### Availability of supporting data

By contact with the corresponding author
